# An individualized risk prediction tool for ectopic pregnancy within the first 10 weeks of gestation based on machine learning algorithms

**DOI:** 10.3389/fmed.2025.1726606

**Published:** 2025-12-09

**Authors:** Xin Du, Qianping Chen, Mengmeng Lu, Jing Hu, Chen Chen, Kaizong Huang, Chunya Ji, Zhou Zhou, Jianjun Zou, Hongjie Ruan

**Affiliations:** 1 Department of Obstetrics and Gynecology, Women’s Hospital of Nanjing Medical University, Nanjing Women and Children’s Healthcare Hospital, Nanjing, China; 2 School of Basic Medicine and Clinical Pharmacy, China Pharmaceutical University, Nanjing, China; 3 Department of Pharmacy, Nanjing First Hospital, Nanjing Medical University, Nanjing, China; 4 Department of Medical Imaging, Women’s Hospital of Nanjing Medical University, Nanjing Women and Children’s Healthcare Hospital, Nanjing, China

**Keywords:** first trimester, pregnancy of unknown location, ectopic pregnancy, machine learning, prediction model

## Abstract

**Background:**

As the main cause of maternal deaths in early pregnancy, delayed diagnosis of ectopic pregnancy (EP) may lead to severe consequences. Patients with pregnancy of unknown location (PUL) exhibit a significantly higher incidence of EP and associated risks compared to the general population. Therefore, this study aims to construct an early prediction model to identify EP risk among patients with PUL and provide a valuable direction for clinical intervention.

**Methods:**

Retrospectively recruited 1896 patients with PUL within 10 weeks of gestation. Feature selection was done using the least absolute shrinkage and selection operator (LASSO). Logistic Regression (LR), Extreme Gradient Boosting (XGB), Random Forest (RFC), Support Vector Machine (SVM), and CatBoost were used to construct the early risk prediction model of EP. The model’s performance was evaluated by the area under the receiver operating characteristic curve (AUROC), the area under the precision-recall curve (AUPRC), and the F1 score. SHapley Additive exPlanations (SHAP) algorithms ranked the feature importance for model output interpretation.

**Results:**

Among the PUL patients included in this study, 66 (4.08%) were diagnosed with EP. Key predictors selected for model construction included vaginal bleeding, progesterone, homogeneous adnexal mass, gravidity, hCG levels, history of cesarean section, abdominal tenderness, and history of pelvic surgery. Among the five models, the CatBoost algorithm demonstrated the best performance, achieving an AUROC of 0.930 (95% CI, 0.876–0.984) and an AUPRC of 0.685 (95% CI, 0.464–0.845). A user-friendly web-based platform was developed for EP risk assessment based on this model. According to SHAP analysis, the three most important clinical predictors were vaginal bleeding, progesterone levels, and the presence of a homogeneous adnexal mass.

**Conclusion:**

This study employed the CatBoost algorithm to develop an individualized risk prediction model by integrating multiple features from the initial visit. This model enhances the detection rate of EP in patients with PUL during early pregnancy. Additionally, we created a web-based tool, offering potential for future clinical applications.

## 1 Introduction

Pregnancy of unknown location (PUL) is characterized by a positive pregnancy test without a definitive embryonic location determined by ultrasound (1). Approximately 4–27% of PUL cases are eventually ultimately confirmed as ectopic pregnancy (EP) (2), a rate significantly higher than the rate compared to the 2% incidence in the general population (3). EP refers to the implantation of an embryo outside the uterine cavity and accounts for approximately 75% of early pregnancy-related deaths (4). Diagnosis typically relies on serial human chorionic gonadotropin (hCG) monitoring and transvaginal ultrasound (TVS) (5–7). However, delayed diagnosis during follow-up can lead to postponed treatment, potentially resulting in severe consequences such as tubal rupture and hemorrhagic shock. While advanced diagnostic techniques such as contrast-enhanced (CE)—magnetic resonance imaging (MRI) (8), intrauterine genomic classifier (9), and biomarkers like miR-519d and sFLT-1 (10, 11) demonstrate promising potential but are restricted by inaccessibility and invasiveness. Consequently, developing convenient, non-invasive, and cost-effective methods for early EP detection remains an urgent priority.

The Society of Obstetricians and Gynaecologists of Canada (SOGC) guidelines on managing PUL recommend utilizing prediction models to facilitate clinical decisions (1). The M4 and M6 are the most extensive models for predicting risk. Both are logistic regression (LR) models that consist of initial serum progesterone, hCG level, and the serum hCG ratio (hCG 48 h/hCG 0 h) (12–15). However, linear models struggle to capture nonlinear relationships among complex clinical factors, impacting risk stratification accuracy. Additionally, low patient compliance and extended follow-up periods increase the risk of adverse outcomes. Machine learning (ML) has proved to be effective for more accurate and personalized medical diagnoses by leveraging large datasets and pattern recognition (16–18). Recent investigations have developed various EP prediction models using ML techniques, exhibiting superior diagnostic performance compared to conventional approaches (19–21). Nonetheless, they are limited by factors such as reliance on single indicators and difficulty in balancing promptness and precision, which restricts their clinical utility.

This study developed an early risk prediction model for PUL by integrating initial clinical assessment data to facilitate triage of high-risk patients. The findings offer a theoretical and methodological foundation for future clinical decision-support tools.

## 2 Methods

### 2.1 Study population

**Figure 1:**
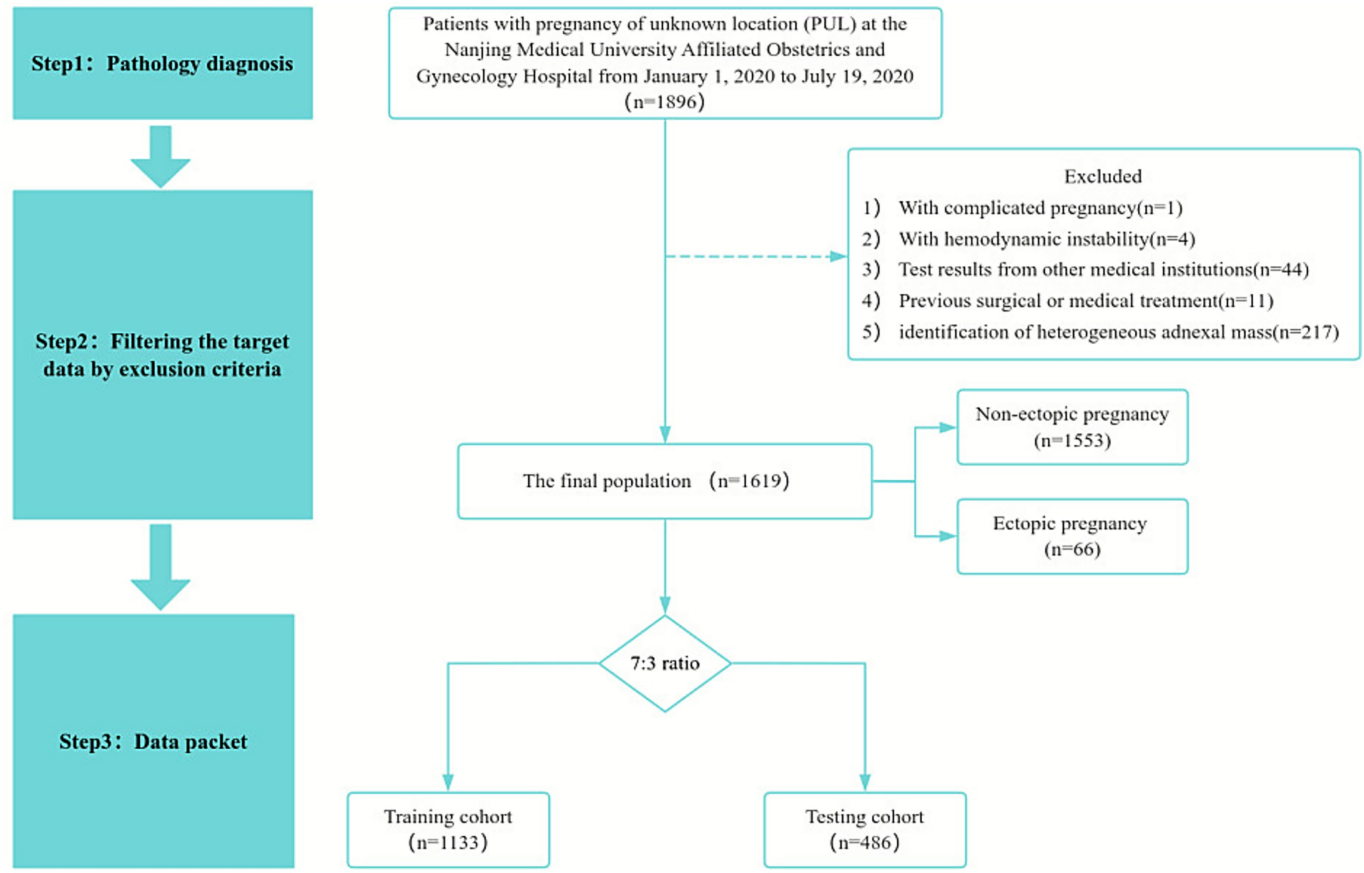
Flow chart of patient enrollment in this study.

This study strictly followed the TRIPOD guidelines (22). The study population consisted of 1,896 patients diagnosed with PUL at the Women’s Hospital of Nanjing Medical University from January 1, 2020 to July 19, 2020. All patients included in the study had a gestational age of less than 10 weeks, presented with biochemically confirmed pregnancy, but lacked an identifiable gestational sac location, which are clinically defined as PUL. Patients were excluded if they had (1) heterotopic pregnancy, (2) hemodynamic instability, (3) test results from other medical institutions, (4) previous surgical or medical treatment, or (5) identification of heterogeneous adnexal mass, which was highly suspicious of EP or adnexal mass of unknown nature. Ethical approval for this study (ID: 2023KY-150) was provided on January 3, 2024, by the Medical Ethics Committee of the Women’s Hospital of Nanjing Medical University (Chair: Yanjing Kan). As the study was retrospective, the ethics committee waived the requirement for informed consent. The flow chart of this study is shown in Figure 1.

### 2.2 Data collection

Clinical data were collected retrospectively from electronic health records in a structured format. In this study, the variables included were baseline characteristics (age, gravidity, parity, abortion), medical history (chronic diseases, gynecological diseases, EP, surgical history, emergency contraceptive pills (ECPs), assisted reproductive technology (ART)), clinical symptoms (abdominal pain, vertigo, diarrhea, abdominal tenderness, cervical motion tenderness, vaginal bleeding), ultrasound findings (homogeneous adnexal mass, pelvic effusion), and serologic marker tests (hCG, progesterone). A homogeneous adnexal mass was defined as an adnexal mass that exhibits uniform internal echogenicity, e.g., cystic or solid masses (23). The serum levels of hCG and progesterone were assessed during the patient’s initial visit. The measurements of hCG and progesterone were conducted in a designated laboratory (Medical Laboratory of Women’s Hospital of Nanjing Medical University) using a fully automated chemiluminescent analyzer and its corresponding reagents (Beckman Coulter, Inc.; Brea, CA, United States). Additionally, in this study, the definition of vaginal bleeding was based on the amount of bleeding compared to menstrual flow, therefore patients were categorized into three groups: no bleeding, bleeding less than, and equivalent menstrual flow.

### 2.3 Sample size calculation

In this research, the sample size of the binary outcome prediction model was calculated according to the following formula: $N=\exp(\frac{-0.508+0.259\ln(\varphi )+0.504\ln(P)-\ln(\text{MAPE})}{0.544})$ (24). In this formula, φ is the anticipated outcome proportion (φ = 0.0408), P is the number of candidate predictor parameters (*p* = 8), and MAPE is the average absolute error between the observed and true outcome probability (MAPE = 0.05). According to calculations, the minimum sample size required for the training set is 145. The total data were randomly divided into the training set and the testing set in a ratio of 7:3, the minimum total effective sample size required is 208.

### 2.4 Outcome

After the entire period of pregnancy follow-up, the final diagnosis was categorized as either EP or non-EP (including normal pregnancy, threatened abortion, spontaneous abortion, missed abortion or inevitable abortion). The outcome of this study was EP, and evidence for diagnosis of EP included (1) ultrasound suggesting the presence of a gestational sac containing a yolk sac or fetal pole (with or without heartbeat) outside the uterine cavity. (2) postoperative pathology confirmation of chorionic villi outside the uterine cavity (1, 4).

### 2.5 Data prepossessing and feature selection

Proper data preprocessing is essential before analysis. In this study, all variables had a completion rate of 100%, except for progesterone (88.2%). Missing values for progesterone were imputed using the k-nearest neighbors (KNN) algorithm (25). KNN fills in missing values by calculating the optimal number of neighbors. To ensure consistency across features, all continuous variables were standardized using z-score normalization, while categorical variables were transformed via one-hot encoding (26). Subsequently, all patients were randomly divided into training and testing cohorts in a 7:3 ratio, ensuring a similar incidence of EP in each cohort. Univariate analysis was performed to identify variables significantly associated with EP (*p* < 0.05). To reduce multicollinearity and improve model performance, the Least Absolute Shrinkage and Selection Operator (LASSO) was employed for feature selection (27). The variance inflation factor (VIF) was subsequently calculated for the selected variables, with VIF < 5 indicating no significant multicollinearity (28).

### 2.6 Model development and evaluation

Five ML algorithms were employed to develop predictive models for EP among patients with PUL: Logistic Regression (LR), Extreme Gradient Boosting (XGB), Random Forest Classifier (RFC), Support Vector Machine (SVM), and CatBoost. Hyperparameters for each model were optimized on the training set using stratified 10-fold cross-validation with a combination of grid search and manual tuning, with the goal of maximizing the area under the precision-recall curve (AUPRC). To preserve the real-world clinical distribution, no oversampling methods (e.g., SMOTE) were applied to balance the outcome classes. Instead, automatic class weight adjustment was utilized during model training. Model performance was evaluated on the testing cohort using primary metrics, including AUPRC, the area under the receiver operating characteristic curve (AUROC), F1 score, sensitivity and specificity. The optimal classification threshold for each model was determined using the Youden index (Youden index = Sensitivity + Specificity − 1). AUROC point estimates, and 95% confidence intervals (CI) were computed using DeLong’s nonparametric method. For AUPRC, sensitivity, specificity, precision and F1-score, 95% CIs were estimated by non-parametric bootstrap resampling (2,000 replicates) using the percentile method. Given the class imbalance in the dataset, AUPRC was emphasized for its sensitivity to positive class performance (29, 30). The F1 score, representing the harmonic mean of precision and recall, was used to summarize model performance at the cut-off. Sensitivity measures the proportion of actual EP cases correctly identified by the model, reflecting its ability to detect high-risk patients. Specificity indicates the proportion of non-EP cases correctly classified, reflecting the model’s ability to avoid unnecessary interventions in low-risk patients. These two metrics are particularly important in imbalanced datasets, where missing a true EP case (false negative) can have severe clinical consequences, while excessive false positives may lead to unnecessary anxiety or diagnostic procedures. Model calibration was measured by the calibration curve and brier score. Calibration curve was plotted to assess how well predicted probabilities matched observed outcomes. The Brier score is the average squared distance between the predicted probability of the outcome and the true label. Decision curve analysis (DCA) was performed to evaluate the clinical usefulness of each prediction model by quantifying the net benefit across a range of threshold probabilities. To improve the stability of estimates, bootstrap resampling (*n* = 1,000) was applied to obtain 95% CIs for each model’s net benefit curve. Based on these comprehensive evaluations, the best-performing model was selected for further interpretation. All models were implemented using Python version 3.12.4, with libraries including Scikit-learn version 1.1.2, XGBoost version 1.0.3, and Keras version 3.5.0.

### 2.7 Model interpretation

The SHapley Additive exPlanations (SHAP) algorithm was employed to enhance the interpretability of the best-performing model, addressing the longstanding “black box” nature of ML models (29). SHAP summary plots were used to display the global importance of each feature based on average absolute SHAP values. Each point on the SHAP scatter plot represents the impact of a given feature on the model’s output for an individual patient, indicating whether the feature increases or decreases the predicted risk of EP. SHAP analysis was performed in Python 3.12.4 using the SHAP package (v0.46.0).

### 2.8 Statistical analysis

Continuous variables were assessed for normality using the Shapiro–Wilk test. Normally distributed data were presented as mean ± standard deviation (SD) and compared using the Student’s *t*-test. Non-normally distributed variables were expressed as median (interquartile range, IQR) and analyzed using the Mann–Whitney U test. Categorical variables were summarized as frequencies and percentages, and comparisons were made using the Chi-square test or Fisher’s exact test, as appropriate. All statistical analyses were performed using R version 4.4.0. Two-tailed *p*-values <0.05 were considered statistically significant.

## 3 Results

### 3.1 Baseline characteristics and outcome

A total of 1,619 patients met the inclusion and exclusion criteria (Figure 1). Baseline characteristics are summarized in Table 1. The median age was 30 years (IQR: 27–33), and 39 patients (2.4%) reported a prior history of EP. Patients were randomly assigned to a training cohort (1,133 patients, 70%) and a testing cohort (486 patients, 30%). No significant differences in baseline characteristics were observed between the two groups (Supplementary Table S1). The overall incidence of EP was 4.08% (66/1,619), with 4.0% (45/1,133) in the training cohort and 4.3% (21/486) in the testing cohort.

**Table 1:** Demographic characteristics of patients in the whole cohort.

Variables	Overall	Non-EP	EP	*P*
	(*n* = 1,619)	*n* = 1,553, 95.92%	*n* = 66, 4.08%	
Demographics				
Age, year	30.00 [27.00, 33.00]	30.00 [27.00, 33.00]	31.50 [28.00, 34.00]	0.144
Gravidity, number of pregnancies	1.00 [0.00, 2.00]	1.00 [0.00, 2.00]	2.00 [0.00, 3.00]	<0.001
Parity, number of deliveries	0.00 [0.00, 1.00]	0.00 [0.00, 1.00]	0.00 [0.00, 1.00]	0.029
Abortion, number of abortions	0.00 [0.00, 1.00]	0.00 [0.00, 1.00]	1.00 [0.00, 2.00]	<0.001
Comorbidities				
History of EP, *n* (%)	39 (2.4)	33 (2.1)	6 (9.1)	0.001
History of laparotomy, *n* (%)	7 (0.4)	7 (0.5)	0 (0.0)	1.000
History of pelvic surgery, *n* (%)	50 (3.1)	41 (2.6)	9 (13.6)	<0.001
History of cesarean section, *n* (%)	171 (10.6)	155 (10.0)	16 (24.2)	<0.001
History of uterine surgery, *n* (%)	14 (0.9)	13 (0.8)	1 (1.5)	1.000
ECPs, *n* (%)	16 (1.0)	14 (0.9)	2 (3.0)	0.281
ART, *n* (%)	29 (1.8)	28 (1.8)	1 (1.5)	1.000
Uterine fibroid, *n* (%)	148 (9.1)	139 (9.0)	9 (13.6)	0.282
Endometriosis, *n* (%)	10 (0.6)	9 (0.6)	1 (1.5)	0.882
Polycystic ovary syndrome, *n* (%)	11 (0.7)	11 (0.7)	0 (0.0)	1.000
Cesarean scar diverticulum, *n* (%)	4 (0.2)	4 (0.3)	0 (0.0)	1.000
Vaginitis, *n* (%)	114 (7.0)	112 (7.2)	2 (3.0)	0.291
PID, *n* (%)	10 (0.6)	7 (0.5)	3 (4.5)	0.001
IUA, *n* (%)	3 (0.2)	2 (0.1)	1 (1.5)	0.270
CUA, *n* (%)	14 (0.9)	12 (0.8)	2 (3.0)	0.207
Cervical polyp, *n* (%)	10 (0.6)	9 (0.6)	1 (1.5)	0.882
Hypertension, *n* (%)	2 (0.1)	2 (0.1)	0 (0.0)	1.000
Diabetes, *n* (%)	3 (0.2)	2 (0.1)	1 (1.5)	0.270
Thyroid diseases, *n* (%)	24 (1.5)	22 (1.4)	2 (3.0)	0.587
Symptoms				
Abdominal pain, *n* (%)	593 (36.6)	565 (36.4)	28 (42.4)	0.386
Vertigo, *n* (%)	3 (0.2)	2 (0.1)	1 (1.5)	0.270
Diarrhea, *n* (%)	5 (0.3)	5 (0.3)	0 (0.0)	1.000
Abdominal tenderness, *n* (%)	24 (1.5)	13 (0.8)	11 (16.7)	<0.001
Cervical motion tenderness, *n* (%)	6 (0.4)	3 (0.2)	3 (4.5)	<0.001
Vaginal bleeding (compare with menstrual flow), *n* (%)				<0.001
None	887 (54.8)	873 (56.2)	14 (21.2)	
Less	657 (40.6)	609 (39.2)	48 (72.7)	
Equivalent	75 (4.6)	71 (4.6)	4 (6.1)	
Ultrasound findings				
Homogeneous adnexal mass, *n* (%)	205 (12.7)	173 (11.1)	32 (48.5)	<0.001
Pelvic effusion, cm	0.00 [0.00, 0.00]	0.00 [0.00, 0.00]	0.00 [0.00, 0.84]	<0.001
Intrauterine echoes, cm	0.58 [0.00, 1.04]	0.60 [0.00, 1.04]	0.00 [0.00, 1.15]	0.354
Serum marker				
hCG, *n* (%)				<0.001
hCG < 1,000, mIU/ml	472 (29.2)	446 (28.7)	26 (39.4)	
1,000 ≤ hCG < 2000, mIU/ml	163 (10.1)	145 (9.3)	18 (27.3)	
2,000 ≤ hCG < 3,000, mIU/ml	124 (7.7)	119 (7.7)	5 (7.6)	
3,000 ≤ hCG < 4,000, mIU/ml	92 (5.7)	91 (5.9)	1 (1.5)	
4,000 ≤ hCG < 5,000, mIU/ml	70 (4.3)	65 (4.2)	5 (7.6)	
hCG ≥ 5,000, mIU/ml	698 (43.1)	687 (44.2)	11 (16.7)	
Progesterone, ng/ml	16.16 [10.60, 21.82]	16.36 [10.97, 21.98]	9.21 [4.82, 14.70]	<0.001

Continuous variables were summarized as median (interquartile range, IQR) and categorical variables were summarized as frequencies and percentages. EP, ectopic pregnancy; ECPs, emergency contraceptive pills; ART, assisted reproductive technology; PID, pelvic inflammatory disease; IUA, intrauterine adhesion; CUA, congenital uterine anomaly; hCG, human chorionic gonadotrophin.

### 3.2 Feature selection and model development

Missing values were imputed using the KNN algorithm, with detailed imputation statistics presented in Supplementary Table S1. Fourteen variables (Table 1) were initially included in the LASSO regression, which ultimately selected eight key predictors: gravidity, vaginal bleeding, hCG, progesterone, homogeneous adnexal mass, history of cesarean section, history of pelvic surgery, and abdominal tenderness. The corresponding non-zero coefficients are provided in Supplementary Table S2. VIF analysis indicated no significant multicollinearity among these predictors (Supplementary Table S3).

### 3.3 Model evaluation and comparison

**Figure 2:**
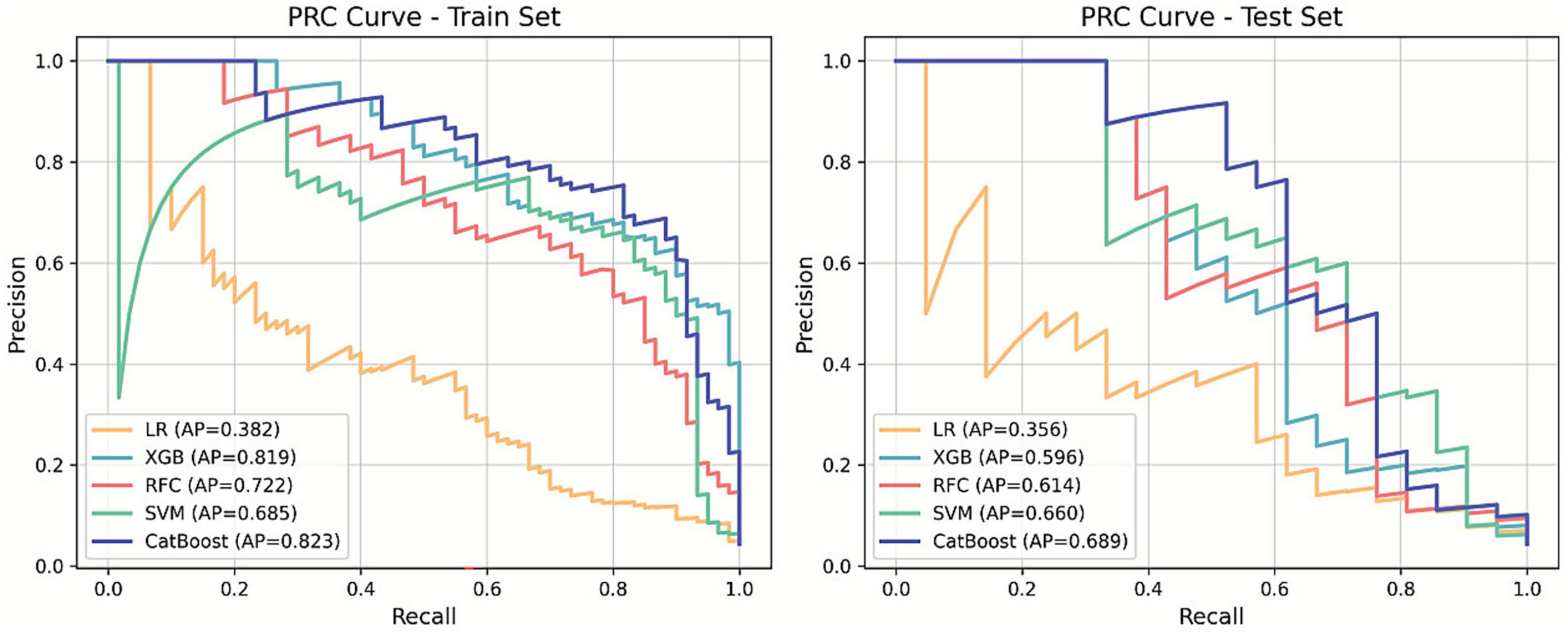
The precision-recall curve (PRC) plots of machine learning predictive models for ectopic pregnancy (EP) in pregnancy of unknown location (PUL) patients.

**Figure 3:**
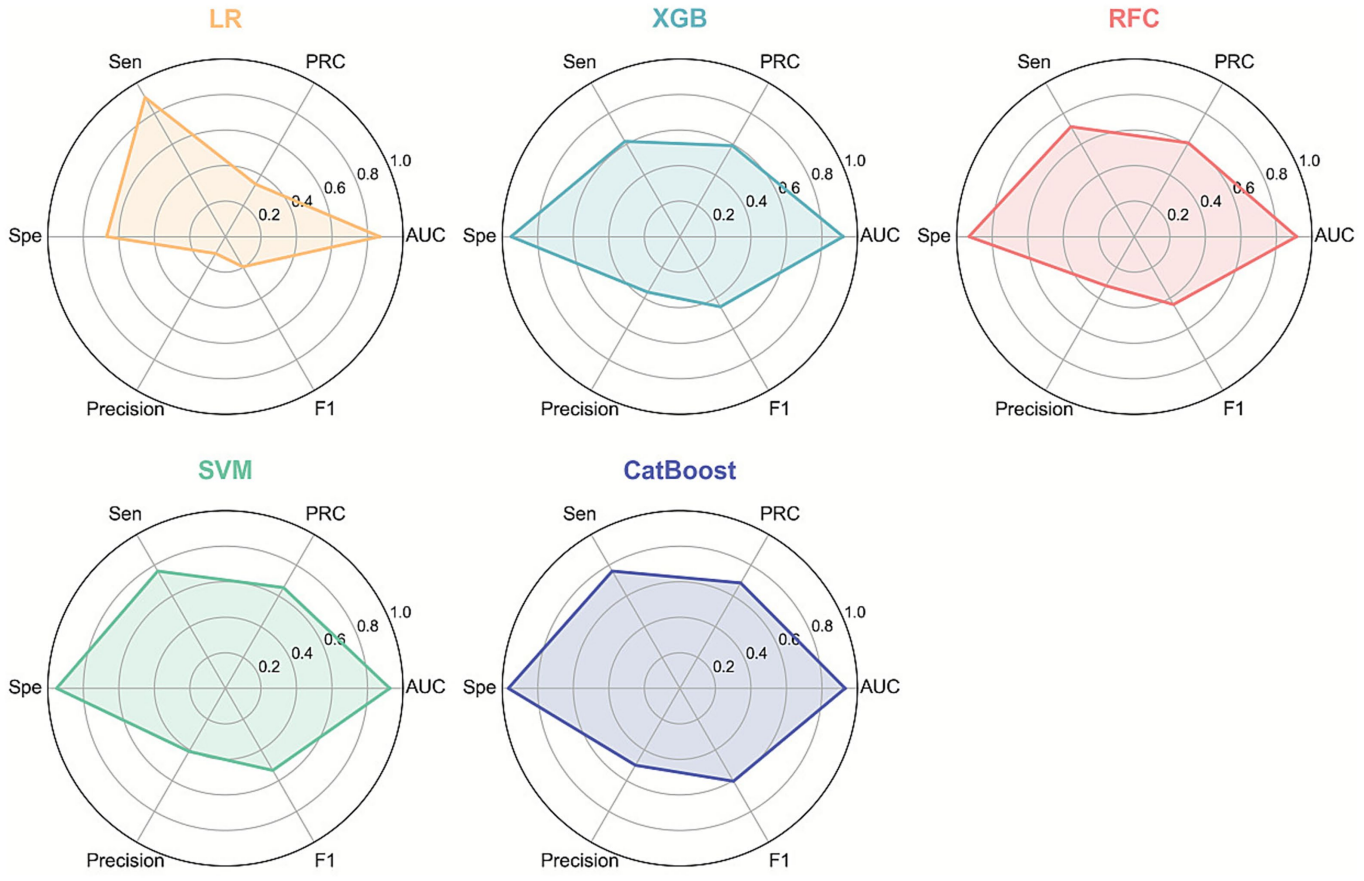
Radar chart comparison of five machine learning models for ectopic pregnancy (EP) prediction. Sen, sensitivity; Spe, specificity.

**Figure 4:**
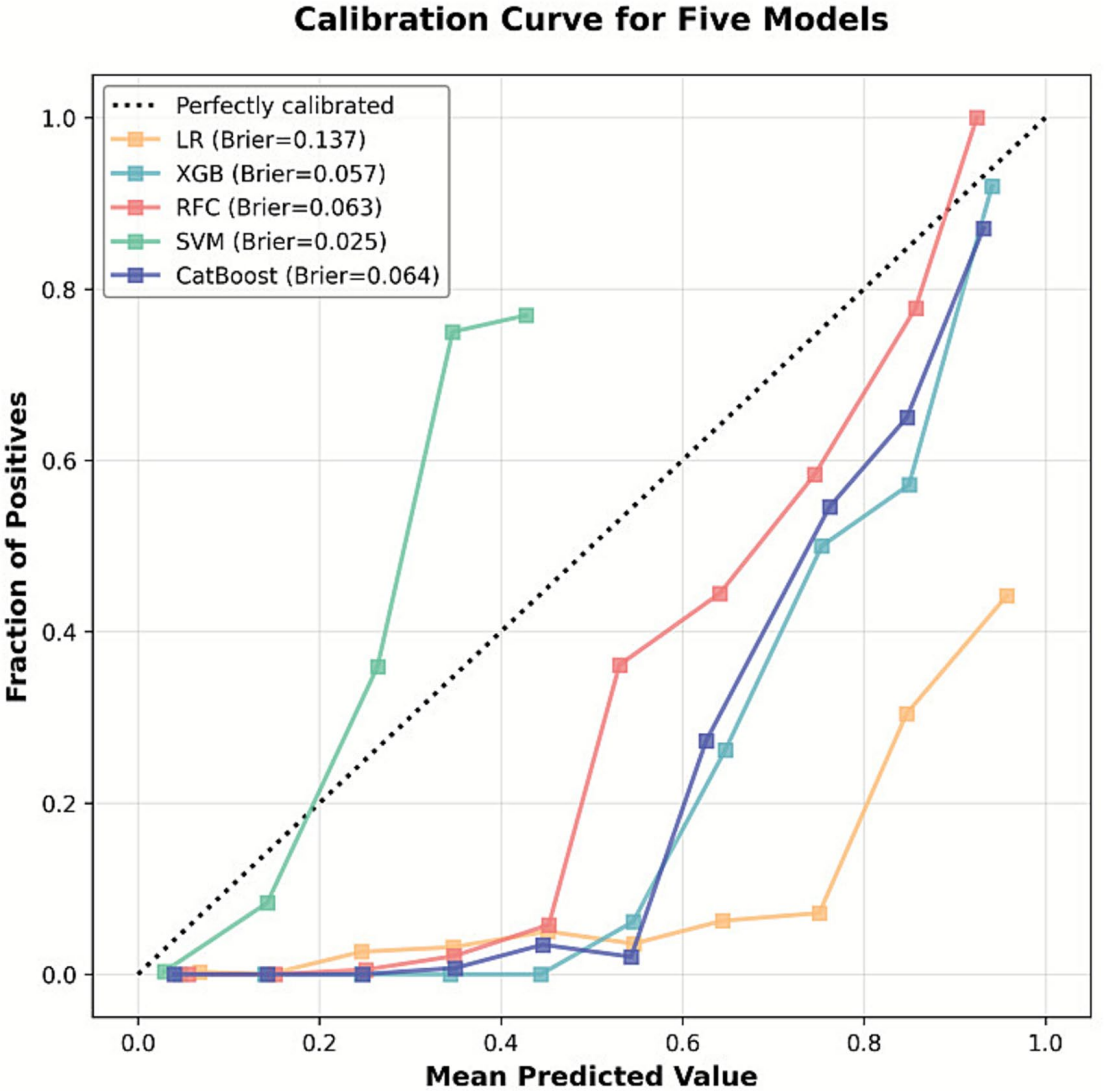
Calibration curve for five machine learning models. The calibration curves depict the agreement between predicted probabilities and actual outcomes in predicting ectopic pregnancy (EP). The diagonal dashed line represents perfect calibration.

**Figure 5:**
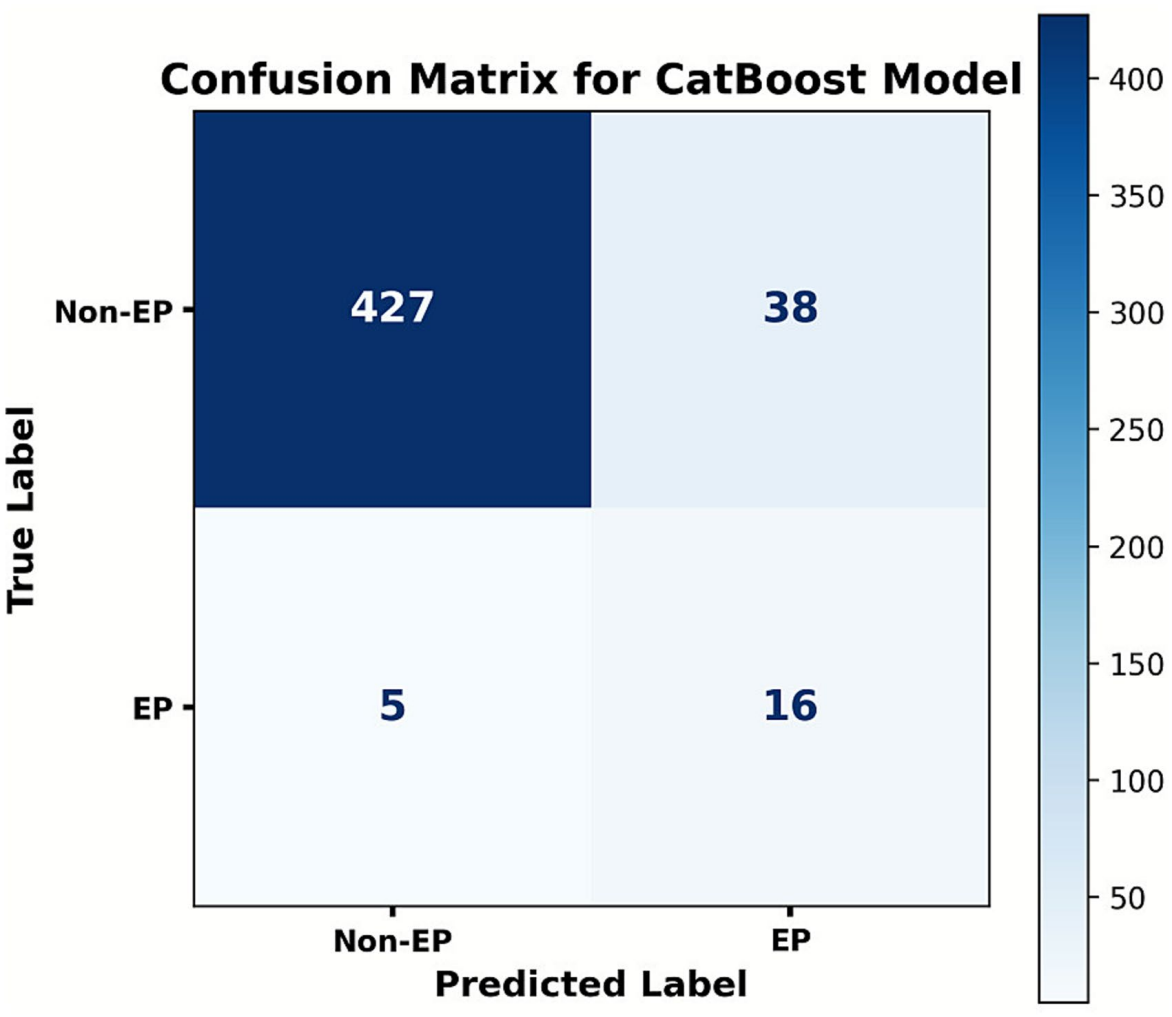
Confusion matrix of the CatBoost model at the cut-off of 0.611. Ordinates represent the actual results, and the abscissa represents the model’s predictive results. The matrix summarizes the case counts of TP (true positive), FN (false positive), FP (false positive), and TN (true negative).

Five ML algorithms—LR, XGB, RFC, SVM, and CatBoost—were trained to predict EP in patients with PUL. Optimal hyperparameter configurations are listed in Supplementary Table S4. Performance metrics are visualized in PRC plots (Figure 2) and radar charts (Figure 3). The cut-off calculated via the Youden Index and the corresponding performance metrics for all models in the testing cohort are presented in Table 2. Among the models, CatBoost achieved the highest performance, with AUPRC of 0.685 (0.493–0.863), AUPRC of 0.930 (0.829–1.000), F1 score of 0.604 (0.432–0.750), sensitivity of 0.762 (0.571–0.944), specificity of 0.966 (0.949–0.981), precision of 0.500 (0.323–0.680), and brier score of 0.064. The calibration curve (Figure 4) showed an acceptable level of calibration for most models, albeit with a tendency to overestimate risk across the predicted probability spectrum. The DCA (Supplementary Figure S1) curve revealed that compared with the “Treat All” and “Treat None” strategies, the CatBoost model provided more clinical utility for EP risk stratification (Supplementary Figure S1). At the cut-off of 0.611, the confusion matrix (Figure 5) on the testing cohort (*n* = 486; EP cases = 21) included 16 true positives, 5 false negatives, 427 true negatives, and 38 false positives (Figure 5). The CatBoost model correctly identified 76.2% of EP cases (16/21). Given the importance of maintaining high sensitivity while limiting false positives in imbalanced datasets, CatBoost demonstrated an effective balance and was selected as the final predictive model.

**Table 2:** Performance of five machine learning models in the testing cohort.

Model	Cut-off	AUROC (95%CI)	AUPRC (95%CI)	F1 score (95%CI)	Sensitivity (95%CI)	Specificity (95%CI)	Precision (95%CI)	Brier score
LR	0.356	0.869 (0.794–0.944)	0.341 (0.173–0.560)	0.196 (0.123–0.275)	0.905 (0.762–1.000)	0.669 (0.627–0.711)	0.110 (0.066–0.160)	0.137
XGB	0.583	0.919 (0.862–0.976)	0.591 (0.377–0.775)	0.456 (0.281–0.603)	0.620 (0.409–0.824)	0.951 (0.930–0.970)	0.361 (0.205–0.526)	0.057
RFC	0.455	0.913 (0.852–0.975)	0.609 (0.393–0.789)	0.441 (0.281–0.571)	0.714 (0.524–0.895)	0.931 (0.909–0.952)	0.319 (0.186–0.453)	0.063
SVM	0.136	0.925 (0.850–1.000)	0.654 (0.435–0.823)	0.533 (0.367–0.678)	0.762 (0.571–0.944)	0.951 (0.930–0.970)	0.410 (0.256–0.575)	0.025
CatBoost	0.611	0.930 (0.829–1.000)	0.685 (0.493–0.863)	0.604 (0.432–0.750)	0.762 (0.571–0.944)	0.966 (0.949–0.981)	0.500 (0.323–0.680)	0.064

LR, logistic regression; XGB, extreme gradient boosting; RFC, random forest classifier; SVM, support vector machine.

### 3.4 Sensitivity analyses

To evaluate the robustness of our findings to different missing value handling strategies, we repeated model development using two alternative imputation methods (median imputation and multivariate imputation by chained equations [MICE]), in addition to the primary k-nearest neighbors (KNN) imputation. Model performance under these three approaches is summarized in Supplementary Tables S5, S6. Results showed no statistically significant differences across imputation strategies (all *p* > 0.05), indicating that our conclusions were not sensitive to the choice of missing value imputation method.

### 3.5 Feature importance visualization

**Figure 6:**
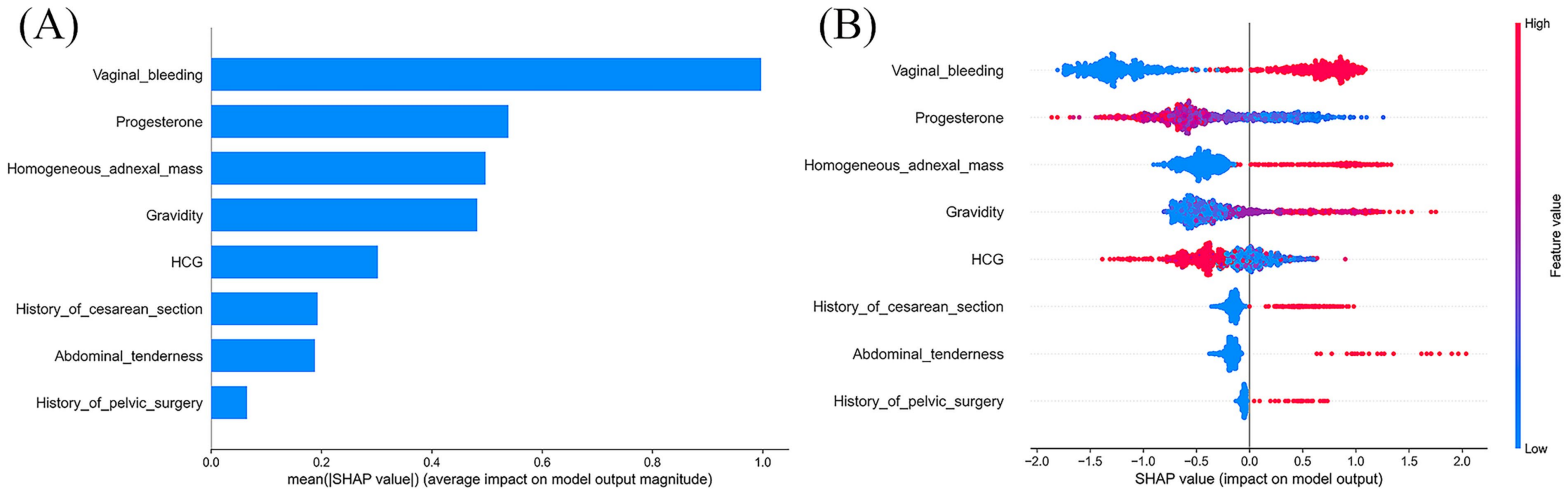
SHAP interpretation of the CatBoost model. **(A)** The importance ranking of each significant predictor; **(B)** Each point represents a feature value. The plot shows the impact of input variables on the CatBoost model’s predictive ability. Red represents a high feature value, whereas blue represents a low one. SHAP, SHapley Additive explanation.

Based on model evaluation, the CatBoost model was confirmed as the optimal predictor. SHAP summary plots (bar and dot) were generated to illustrate feature contributions (Figure 6). Vaginal bleeding, progesterone, and homogeneous adnexal mass were the top three predictors based on SHAP values. To further explore potential non-linear effects of individual predictors, we plotted univariable SHAP dependence plots (Supplementary Figure S2). The SHAP dependence plot indicated that less vaginal bleeding (relative to menstrual flow), lower progesterone levels and presence of a homogeneous adnexal mass were associated with increased EP risk. Additionally, a history of cesarean section or pelvic surgery further elevated EP risk in the model’s predictions.

### 3.6 Web-based prediction platform

**Figure 7:**
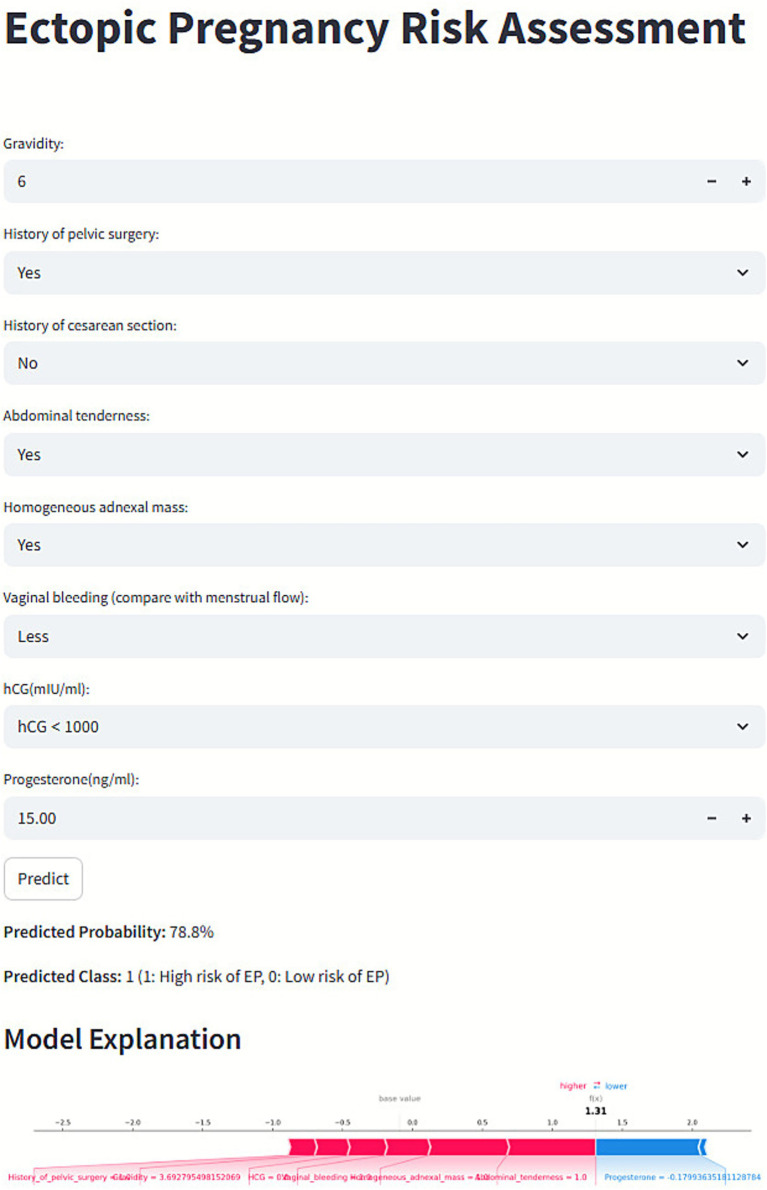
Web-based prediction platform for ectopic pregnancy (EP) risk assessment.

To facilitate clinical application, a user-friendly web platform (Figure 7) was developed for EP risk prediction: https://kclba5qc7vr7gyoa8kjakj.streamlit.app/. Users can input relevant clinical data to receive a personalized EP risk prediction, categorized as low (0) or high (1) risk. The platform also provides SHAP-based visual explanations, including force plots, to highlight key factors influencing individual predictions.

## 4 Discussion

In this study, we established an early prediction model to identify EP risk among patients with PUL, utilizing five ML algorithms, including LR, XGB, RFC, SVM, and CatBoost. Among them, the CatBoost model had the highest performance, with a PRC of 0.685 (95% CI, 0.464–0.845), a sensitivity of 76.2%, a specificity of 96.6%, and an F1 score of 0.604. Feature interpretation was conducted using SHAP, which enabled ranking the importance of eight predictors. The top five clinical factors contributing to EP risk were: the amount of vaginal bleeding, serum progesterone level, presence of adnexal mass, gravidity, and hCG concentration.

Innovatively employing baseline clinical data, the CatBoost model helped clinicians complete early EP risk stratification at the patient’s initial visit. Conventionally, previous models (30, 31) relied on serial hCG monitoring over 48 h to 7 days, which might delay clinical decisions and compromise patient compliance. From a pathophysiological perspective, trophoblast dysfunction in EP patients stemmed from abnormal embryo implantation, leading to significantly lower early hormone levels than normal intrauterine pregnancies (32, 33). Therefore, baseline hCG and progesterone, as direct indicators of trophoblast development, were valuable for initial EP risk assessment (1). To some extent, the model aided in preventing delayed intervention in high-risk patients and mitigated the risk of fatal complications, such as tubal rupture and hemorrhagic shock, in regions with constrained healthcare resources or high patient mobility, this model had the potential to minimize the probability of missed diagnosis and loss to follow-up, thereby improving diagnostic timeliness and clinical accessibility.

Notably, this study focused on PUL patients within the first 10 weeks of gestation, rather than the traditional 14-week definition (34). During early pregnancy, hCG levels followed a characteristic trajectory: serum *β*-hCG became detectable approximately 10 days post-fertilization, rose exponentially around week 5, peaked at 9–10 weeks, and then gradually declined to a plateau in the second and third trimesters (35, 36). Early hCG levels were critical for umbilical cord development, inhibition of uterine contractions, fetal organogenesis, angiogenesis, and immune tolerance regulation (37). Therefore, we selected a predictive time window within the first 10 weeks to capture subtle fluctuations in early hCG dynamics and evaluate relevant risk factors.

Through the integration of multiple features, this study investigated the synergistic predictive value of baseline serum biomarkers and clinical symptoms to better understand disease progression and optimize clinical management in the PUL population. We found that higher initial hCG and progesterone levels did not indicate an increased risk of EP. Conversely, EP patients generally exhibited lower levels compared to non-EP individuals. From a pathophysiological perspective, implantation in EP occurred outside the uterus, predominantly in the fallopian tube. When the trophoblast was in a suboptimal environment, hCG and progesterone synthesis and secretion might reduce, leading to hormonal imbalance. The hormonal imbalance impaired endometrial maintenance, this could cause decidual tissue breakdown and shedding, which typically manifested clinically as vaginal bleeding or spotting (38, 39). This was why irregular vaginal bleeding, especially when bleeding volume was less than normal menstrual blood loss, might suggest a higher risk of EP in PUL patients. Interestingly, the SHAP revealed that the contribution of vaginal bleeding to EP risk prediction was higher than that of hCG and progesterone levels. Meanwhile, several previous studies corroborated our findings (40–42). Moreover, while gravidity is a well-established risk factor, its confounding associations with other characteristics must also be considered, as they may significantly influence outcome prediction.

Despite the low prevalence of EP in the PUL population (4.08%), relying on the multidimensional optimization of sensitivity, specificity, and precision, the model achieved good predictive performance. Specifically, the 96.6% specificity effectively excluded non-EP cases, thereby minimizing unnecessary examinations, conserving medical resources, and alleviating patient anxiety. Although the model’s sensitivity (76.2%) was lower than that of the M4 (80.0–86.4%) and M6NP (90.6–95.0%) models (12–15, 43, 44), it still captured over 75% of potential EP cases, providing a critical time window for early intervention. Additionally, our CatBoost model achieved a precision of 50%, which was higher than that of the M6NP model (18.7%) and the low-accuracy subgroup of the M4 model (37.8%). Notably, although the LR model demonstrated superior sensitivity, the CatBoost model achieved a more reasonable balance between sensitivity and precision. In EP prediction, calibration curves evaluating the CatBoost model revealed systematic overestimation. Given that the primary objective of this study was to achieve early identification of potential EP cases and thereby prevent missed diagnoses that might lead to life-threatening complications. Thus, prioritization of higher predicted risk was considered preferable to underestimation. This approach aids in identifying high-risk patients and provides valuable guidance for clinical intervention. Notably, DCA demonstrated that the model’s net benefit within the threshold probability range commonly used in clinical practice was significantly superior to both the “Treat All” and “Treat None” strategies.

False-negative and false-positive results were used to assist clinicians in identifying the causes of model prediction bias, thereby improving the accuracy and safety of EP diagnosis. In the CatBoost model’s testing set, 5 false-negative cases were associated with hCG and progesterone levels at diagnostic cutoffs, absence of typical ultrasonographic findings, and atypical early EP symptoms. Meanwhile, 38 false-positive cases were attributed to three core factors: highly analogous clinical phenotypes, comorbidity interference, and predictive feature interactions. For instance, concurrent benign adnexal masses or pelvic inflammatory disease were erroneously captured as positive signals, resulting in false positives. Therefore, given the complexity of EP pathogenesis and the limitations of early clinical detection methods, prediction errors were inevitable. To address this issue, the AUPRC was chosen as a more suitable evaluation metric (45). By balancing precision and recall, the AUPRC minimized the risk of missed diagnoses and false positives, ensuring the model’s predictive performance was both reliable and stable. Importantly, the model was intended as a clinical decision-support tool, not as a replacement for clinicians’ professional judgment. It provided objective risk assessment references to aid clinicians in diagnosing and making decisions about EP. Despite unavoidable misclassifications, the model demonstrated strong overall performance (AUROC: 0.930, AUPRC: 0.685) and remained viable for widespread clinical use.

Nevertheless, this study has several limitations. Firstly, as a single-center retrospective analysis, the demographic characteristics of the study population were relatively homogeneous. Previous studies had shown that populations from different regions and social backgrounds demonstrate substantial variations in the incidence and prognosis of EP (46, 47). Secondly, the number of positive cases was limited. However, methodological optimizations were applied to partially mitigate its potential impact. Thirdly, as this study centered on initial-visit assessments, serial *β*-hCG measurements were not incorporated. Finally, certain characteristics were absent, including the lack of EP cases occurring in rare locations such as the interstitial segment or ovary, which could result in severe outcomes when diagnosis was delayed (48, 49). Furthermore, due to sample size limitations, the model incorporated neither unobserved nor undocumented symptoms from our patient cohort. Future multi-center prospective studies are planned to have the sample size expanded, a more diverse patient cohort enrolled, and comprehensive baseline clinical data collected, including dynamic serological markers and multidimensional clinical symptoms, thereby enhancing the external validity and robustness of the developed model.

## 5 Conclusion

We have devised a personalized risk prediction model utilizing the CatBoost algorithm to facilitate the early detection of EP within the PUL population. This model incorporates eight crucial predictors including vaginal bleeding, progesterone level, and adnexal mass. It differentiates non-EP cases and identifies potential EP cases. The importance of these predictors was illustrated through SHAP summary plots. Furthermore, a web platform was created as a proof-of-concept tool, setting the stage for the rigorous external validation and prospective studies prior to clinical application.

## Data Availability

The original contributions presented in the study are included in the article/Supplementary material, further inquiries can be directed to the corresponding authors.
