# Supplementary material for: An individualized risk prediction tool for ectopic pregnancy within the first 10 weeks of gestation based on machine learning algorithms
Source: Front Med (Lausanne). 2025 Dec 9;12:1726606. doi: 10.3389/fmed.2025.1726606 (PMC12722958; doi:10.3389/fmed.2025.1726606)
Supplement: Supplementary file 1 [file Data_Sheet_1.docx]

Supplementary Material

# Supplementary Figures and Tables

## Supplementary Figures


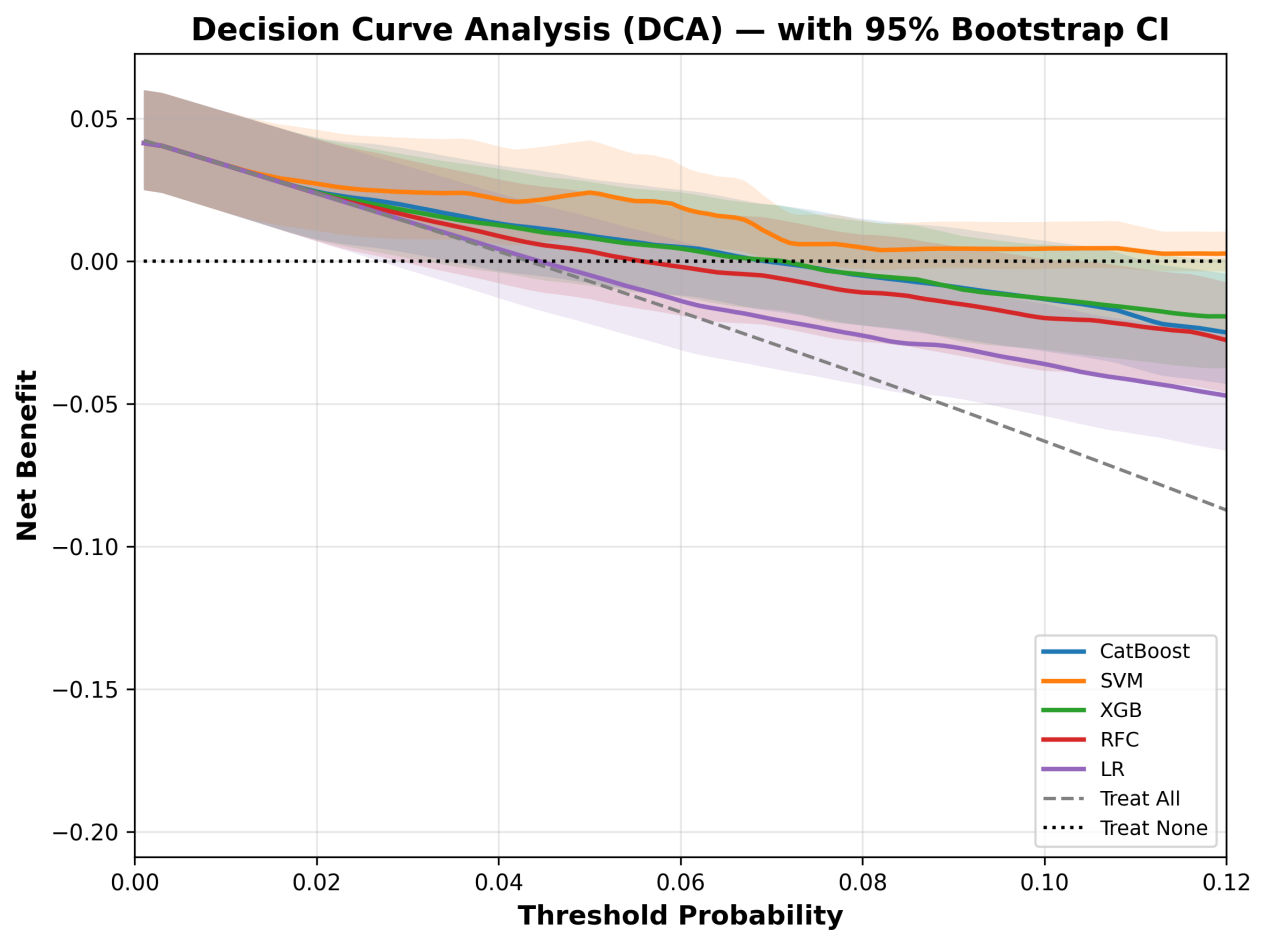


**Supplementary Figure S1.** Decision curve analysis (DCA) of five models for ectopic pregnancy prediction.


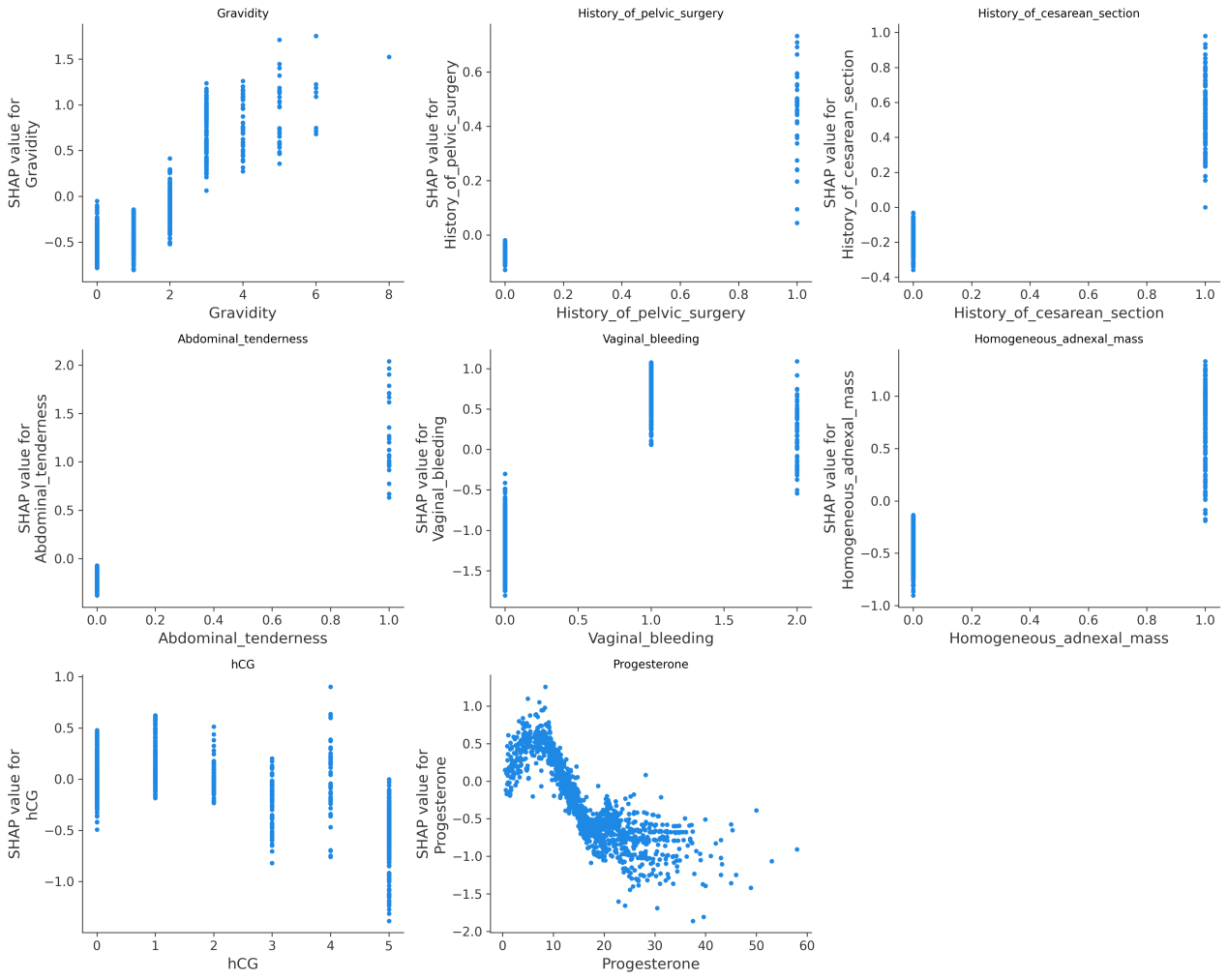


**Supplementary Figure S2.** Univariate SHAP dependence plot. For binary variables: "0" represents "No" and "1" represents "Yes" for history of pelvic surgery, history of cesarean section, abdominal tenderness, and homogeneous adnexal mass. For vaginal bleeding, "0", "1", and "2" correspond to "no bleeding", "bleeding less than menstrual volume", and "bleeding equal to menstrual volume", respectively. For hCG, the categories "1-5" denote ranges of hCG<1000, 1000-2000, 2000-3000, 3000-4000, 4000-5000, and≥5000mIU/ml

## Supplementary Tables

**Supplementary Table S1.** Comparison of the characteristics of patients between the training cohort and the testing cohort.

| **Variables** | Overall | Training cohort | Testing cohort | P | Missing rate |
| --- | --- | --- | --- | --- | --- |
|  | (n=1619) | (n=1133) | (n=486) |  |  |
| **Demographics** |  |  |  |  |  |
| Age, year | 30.00 [27.00, 33.00] | 30.00 [28.00, 33.00] | 30.00 [27.00, 33.00] | 0.389 | 0% |
| Gravidity, number of pregnancies | 1.00 [0.00, 2.00] | 1.00 [0.00, 2.00] | 1.00 [0.00, 2.00] | 0.873 | 0% |
| Parity, number of deliveries | 0.00 [0.00, 1.00] | 0.00 [0.00, 1.00] | 0.00 [0.00, 1.00] | 0.743 | 0% |
| Abortion, number of abortions | 0.00 [0.00, 1.00] | 0.00 [0.00, 1.00] | 0.00 [0.00, 1.00] | 0.738 | 0% |
| **Comorbidities** |  |  |  |  |  |
| History of ectopic pregnancy, n (%) | 39 (2.4) | 26 (2.3) | 13 (2.7) | 0.779 | 0% |
| History of laparotomy, n (%) | 7 (0.4) | 5 (0.4) | 2 (0.4) | 1.000 | 0% |
| History of pelvic surgery, n (%) | 50 (3.1) | 31 (2.7) | 19 (3.9) | 0.274 | 0% |
| History of cesarean section, n (%) | 171 (10.6) | 124 (10.9) | 47 (9.7) | 0.499 | 0% |
| History of uterine surgery, n (%) | 14 (0.9) | 9 (0.8) | 5 (1.0) | 0.862 | 0% |
| ECPs, n (%) | 16 (1.0) | 9 (0.8) | 7 (1.4) | 0.352 | 0% |
| ART, n (%) | 29 (1.8) | 20 (1.8) | 9 (1.9) | 1.000 | 0% |
| Uterine fibroid, n (%) | 148 (9.1) | 98 (8.6) | 50 (10.3) | 0.340 | 0% |
| Endometriosis, n (%) | 10 (0.6) | 9 (0.8) | 1 (0.2) | 0.299 | 0% |
| Polycystic ovary syndrome, n (%) | 11 (0.7) | 7 (0.6) | 4 (0.8) | 0.896 | 0% |
| Vaginitis, n (%) | 114 (7.0) | 87 (7.7) | 27 (5.6) | 0.154 | 0% |
| Cesarean scar diverticulum, n (%) | 4 (0.2) | 2 (0.2) | 2 (0.4) | 0.744 | 0% |
| PID, n (%) | 10 (0.6) | 7 (0.6) | 3 (0.6) | 1.000 | 0% |
| IUA, n (%) | 3 (0.2) | 2 (0.2) | 1 (0.2) | 1.000 | 0% |
| CUA, n (%) | 14 (0.9) | 8 (0.7) | 6 (1.2) | 0.447 | 0% |
| Cervical polyp, n (%) | 10 (0.6) | 6 (0.5) | 4 (0.8) | 0.730 | 0% |
| Hypertension, n (%) | 2 (0.1) | 1 (0.1) | 1 (0.2) | 1.000 | 0% |
| Diabetes, n (%) | 3 (0.2) | 2 (0.2) | 1 (0.2) | 1.000 | 0% |
| Thyroid diseases, n (%) | 24 (1.5) | 15 (1.3) | 9 (1.9) | 0.561 | 0% |
| **Symptoms** |  |  |  |  |  |
| Abdominal pain, n (%) | 593 (36.6) | 407 (35.9) | 186 (38.3) | 0.399 | 0% |
| Vertigo, n (%) | 3 (0.2) | 2 (0.2) | 1 (0.2) | 1.000 | 0% |
| Diarrhea, n (%) | 5 (0.3) | 2 (0.2) | 3 (0.6) | 0.329 | 0% |
| Abdominal tenderness, n (%) | 24 (1.5) | 15 (1.3) | 9 (1.9) | 0.561 | 0% |
| Cervical motion tenderness, n (%) | 6 (0.4) | 4 (0.4) | 2 (0.4) | 1.000 | 0% |
| Vaginal bleeding (compare with menstrual flow), n (%) |  |  |  | 0.848 | 0% |
| None | 887 (54.8) | 626 (55.3) | 261 (53.7) |  |  |
| Less | 657 (40.6) | 455 (40.2) | 202 (41.6) |  |  |
| Equivalent | 75 (4.6) | 52 (4.6) | 23 (4.7) |  |  |
| **Ultrasound findings** |  |  |  |  |  |
| Homogeneous adnexal mass, n (%) | 205 (12.7) | 145 (12.8) | 60 (12.3) | 0.866 | 0% |
| Pelvic effusion,cm | 0.00 [0.00, 0.00] | 0.00 [0.00, 0.00] | 0.00 [0.00, 0.00] | 0.398 | 0% |
| Intrauterine echoes,cm | 0.58 [0.00, 1.04] | 0.60 [0.00, 1.03] | 0.58 [0.00, 1.07] | 0.852 | 0% |
| **Serum marker** |  |  |  |  |  |
| hCG, n (%) |  |  |  | 0.842 | 0% |
| hCG<1000, mIU/ml | 472 (29.2) | 328 (28.9) | 144 (29.6) |  |  |
| 1000≤hCG<2000, mIU/ml | 163 (10.1) | 116 (10.2) | 47 (9.7) |  |  |
| 2000≤hCG<3000, mIU/ml | 124 (7.7) | 86 (7.6) | 38 (7.8) |  |  |
| 3000≤hCG<4000, mIU/ml | 92 (5.7) | 65 (5.7) | 27 (5.6) |  |  |
| 4000≤hCG<5000, mIU/ml | 70 (4.3) | 54 (4.8) | 16 (3.3) |  |  |
| hCG≥5000, mIU/ml | 698 (43.1) | 484 (42.7) | 214 (44.0) |  |  |
| Progesterone, ng/ml | 16.16 [10.60, 21.82] | 16.21 [10.57, 21.89] | 15.96 [10.88, 21.70] | 0.714 | 11.8% |
| **Outcome** |  |  |  |  |  |
| EP, n (%) | 66 (4.1) | 45 (4.0) | 21 (4.3) | 0.850 | 0% |
| Continuous variables were summarized as median (interquartile range, IQR) and categorical variables were summarized as frequencies and percentages. EP, ectopic pregnancy; ECPs, emergency contraceptive pills; ART, assisted reproductive technology; PID, pelvic inflammatory disease; IUA, intrauterine adhesion; CUA, congenital uterine anomaly; hCG, human chorionic gonadotrophin | | | | | |

**Supplementary Table S2.** The detailed coefficients of all non-zero coefficient variables after LASSO.

| **Variables** | **coefficient** |
| --- | --- |
| Gravidity | 0.1550 |
| Vaginal bleeding | 0.4924 |
| hCG | -0.0221 |
| Progesterone | -0.0270 |
| Homogeneous adnexal mass | 1.1748 |
| History of cesarean section | 0.2575 |
| History of pelvic surgery | 0.8995 |
| Abdominal tenderness | 1.8050 |
| LASSO, the least absolute shrinkage and selection operator; hCG, human chorionic gonadotrophin | |

**Supplementary Table S3.** Collinearity analysis of the eight predictive variables.

| **Variables** | **VIF** |
| --- | --- |
| Gravidity | 1.182 |
| Vaginal bleeding | 1.065 |
| hCG | 1.089 |
| Progesterone | 1.074 |
| Homogeneous adnexal mass | 1.016 |
| History of cesarean section | 1.158 |
| History of pelvic surgery | 1.038 |
| Abdominal tenderness | 1.038 |
| VIF, Variance Inflation Factor; hCG, human chorionic gonadotrophin. | |

**Supplementary Table S4.** Optimal hyperparameters for each model.

| Classifier models | Hyper-parameter | Optimal value |
| --- | --- | --- |
| LR | C | 4 |
|  | Penalty | L1 |
|  | Solver | liblinear |
|  | Class weight | balanced |
|  |  |  |
| XGB | N estimators | 480 |
|  | Learning rate | 0.01 |
|  | Max depth | 6 |
|  | Gamma | 0.1 |
|  | Min child weight | 8 |
|  | Colsample bytree | 0.6 |
|  | Subsample | 0.8 |
|  | Scale pos weight | sum(trainy==0) / sum(trainy==1) |
|  |  |  |
| RFC | N estimators | 450 |
|  | Max depth | 6 |
|  | Min samples leaf | 2 |
|  | Min samples split | 5 |
|  | Class weight | balanced |
|  |  |  |
| SVM | Kernel | rbf |
|  | C | 0.06 |
|  | Gamma | 1.5 |
|  | Class weight | balanced |
|  |  |  |
| CatBoost | Iterations | 550 |
|  | Depth | 8 |
|  | Learning rate | 0.005 |
|  | L2 leaf reg | 5 |
|  | Bagging temperature | 3 |
|  | Class weight | balanced |

LR, logistic regression; XGB, extreme gradient boosting; RFC, random forest classifier; SVM, support vector machine.

**Supplementary Table S5.** Model performance under three different missing value imputation methods

| **Method** | **AUROC (95%CI)** | **AUPRC (95%CI)** | **F1 score(95%CI)** | **Sensitivity(95%CI)** | **Specificity(95%CI)** | **Precision(95%CI)** |
| --- | --- | --- | --- | --- | --- | --- |
| KNN | 0.930(0.829-1.000) | 0.685(0.493-0.863) | 0.604 (0.432-0.750) | 0.762 (0.571-0.944) | 0.966(0.949-0.981) | 0.500 (0.323-0.680) |
| Median | 0.916(0.805-1.000) | 0.683(0.495-0.851) | 0.478(0.320-0.615) | 0.762 (0.571-0.944) | 0.936 (0.914-0.957) | 0.348(0.211-0.489) |
| MICE | 0.921(0.812-1.000) | 0.647(0.457-0.820) | 0.571(0.400-0.712) | 0.762 (0.571-0.944) | 0.959(0.940-0.976) | 0.457(0.296-0.625) |
| KNN：K-Nearest Neighbors；MICE：Multivariate Imputation by Chained Equations | | | | | | |

**Supplementary Table S6.** Sensitivity analysis of missing value imputation methods

| Compare | Metric | Diff | 95%CI | p |
| --- | --- | --- | --- | --- |
| KNN vs Median | AUC | 0.010 |  | 0.905 |
|  | PRC | 0.089 | (-0.187, 0.356) | 0.554 |
|  | F1 | 0.061 | (-0.164, 0.282) | 0.585 |
|  | Sensitivity | 0.000 | (-0.248, 0.267) | 0.976 |
|  | Specificity | 0.013 | (-0.011, 0.039) | 0.301 |
|  | Precision | 0.079 | (-0.156, 0.308) | 0.505 |
| KNN vs MICE | AUC | 0.010 |  | 0.899 |
|  | PRC | 0.041 | (-0.226, 0.298) | 0.772 |
|  | F1 | 0.032 | (-0.185, 0.253) | 0.762 |
|  | Sensitivity | 0.000 | (-0.248, 0.267) | 0.976 |
|  | Specificity | 0.006 | (-0.017, 0.032) | 0.579 |
|  | Precision | 0.043 | (-0.196, 0.280) | 0.717 |
| KNN; K-Nearest Neighbors; MICE: Multivariate Imputation by Chained Equations | | | | |
